# Therapeutic Dilemma in Epidural Catheter Removal During Dual Antiplatelet Therapy and Anticoagulation Following Hepatic Artery Injury: A Case Report

**DOI:** 10.7759/cureus.113117

**Published:** 2026-07-21

**Authors:** Talita Larissa de Castro Lousada, Ana Paula de Souza Barbosa Ferreira, Bruno Eduardo Silva Dorna, Lucas Augusto Carvalho e Raso

**Affiliations:** 1 Medical School, University of Ribeirão Preto (UNAERP), Guarujá, BRA; 2 Anesthesia, Universidade Federal de Uberlândia, Uberlândia, BRA

**Keywords:** asra guidelines, case report, drug eluting stent, dual anti-platelet therapy, epidural analgesia, epidural analgesia complications

## Abstract

Epidural catheter removal in the setting of concurrent dual antiplatelet therapy (DAPT) and anticoagulation poses a high risk of neuraxial hematoma. Current guidelines mandate prolonged interruption of antithrombotic therapy, which may be unfeasible in patients with recently implanted arterial stents at high risk of thrombosis. We present the case of a 56-year-old man who underwent combined epidural and general anesthesia for a partial hepatectomy. An inadvertent intraoperative injury to the proper hepatic artery required aborting the surgery and deployment of a rescue drug-eluting stent. Intravenous unfractionated heparin and DAPT (aspirin and clopidogrel) were initiated. On postoperative day 7, inflammatory signs at the epidural catheter site made its removal urgent. However, interrupting clopidogrel for the guideline-recommended five to seven days posed an unacceptable risk of acute thrombosis of the newly deployed hepatic artery stent. To navigate this conflict between preventing neuraxial hematoma and acute hepatic ischemia, a multidisciplinary team reached a consensus to implement an unorthodox 24-hour therapeutic window off antithrombotics. The catheter was removed uneventfully at the end of this period; antiplatelet therapy and enoxaparin were restarted at two and six hours, respectively. No neurological deficits occurred. This case illustrates that, in scenarios of competing high-risk priorities, strict adherence to standardized recommendations may not be feasible. An individualized strategy based on multidisciplinary consensus, careful timing of antithrombotic interruption and reintroduction, and close neurological monitoring was successfully implemented without complications in this patient. However, given the limitations of a single case report, this individualized salvage strategy should not be generalized and requires further clinical evidence.

## Introduction

Postoperative pain following open hepatectomy is substantial and mandates a well-structured analgesic strategy. The 2021 Procedure-Specific Postoperative Pain Management (PROSPECT) guidelines reviewed 31 randomized controlled trials and three systematic reviews regarding pain management in open liver resection [1]. While emphasizing opioid-sparing multimodal analgesia, the consensus maintains continuous thoracic epidural analgesia as a cornerstone recommendation for this procedure.

Despite meticulous perioperative management, liver resections carry an inherent risk of hemorrhage and coagulopathy, complicating the optimal timing of postoperative epidural catheter removal [2]. However, the occurrence of rare, catastrophic adverse events - such as a major hepatic artery injury - dramatically escalates clinical complexity. Subsequent vascular repair and stent angioplasty mandate systemic heparinization and uninterrupted dual antiplatelet therapy (DAPT) during the immediate postoperative period.

This exceptionally rare scenario presents a therapeutic dilemma. Prolonged catheter retention poses a severe risk of infection, precluding the indefinite maintenance of the invasive device. Conversely, current regional anesthesia guidelines, such as those from the American Society of Regional Anesthesia and Pain Medicine (ASRA), contraindicate epidural catheter manipulation or removal in the setting of concurrent heparinization and DAPT due to the high risk of neuraxial hematoma, and they do not offer specific recommendations for these exceptional situations [3]. Thus, this article reports the challenging multidisciplinary decision-making and management of a patient who required epidural catheter removal without standard discontinuation of antithrombotic therapy, illustrating the need to individualize care when established protocols conflict.

## Case presentation

A 56-year-old male patient with no significant comorbidities and a hepatic tumor located in segment VI was admitted for an open partial hepatectomy. The planned anesthetic technique consisted of a thoracic epidural with catheter (at the T8-T9 level) followed by general anesthesia. During the initial dissection of the hepatic hilum in preparation for the Pringle maneuver, an inadvertent injury to the proper hepatic artery occurred. Intravenous unfractionated heparin (5,000 IU) was administered, and the injury was repaired. The oncological resection was aborted, and the patient was transferred to the ICU with a patient-controlled epidural analgesia pump. Shortly after admission, absence of hepatic arterial flow was identified through Doppler ultrasound and computed tomography angiography. He was immediately transferred to the catheterization laboratory, where vascular repair was complemented via the deployment of a drug-eluting stent. To maintain stent patency, continuous DAPT (aspirin 100 mg and clopidogrel 75 mg) was initiated without a loading dose. Concurrently, subcutaneous unfractionated heparin was initiated for the prevention of deep vein thrombosis (5,000 IU every eight hours). On the fifth postoperative day, unfractionated heparin was transitioned to low-molecular-weight heparin (LMWH), specifically enoxaparin 40 mg subcutaneously once daily. A detailed chronological breakdown of the clinical course and medication timelines is summarized in Table 1.

**Table 1:** Chronological timeline of perioperative events, antithrombotic therapy, and clinical interventions ICU: intensive care unit; CT: computed tomography; DAPT: dual antiplatelet therapy; PCEA: patient-controlled epidural analgesia; IU: international units; DVT: deep vein thrombosis; q8h: every 8 hours; q4h: every 4 hours; q2h: every 2 hours; QD: once daily

Timepoint	Clinical events	Antithrombotic/anticoagulant therapy	Epidural catheter management
Day 0 (Intraoperative)	Open partial hepatectomy (aborted); inadvertent proper hepatic artery injury and immediate vascular repair.	5,000 IU intravenous unfractionated heparin administered intraoperatively.	Thoracic epidural catheter placed pre-induction (T8-T9 level).
Day 0 (Postoperative)	Transfer to ICU; Doppler ultrasound and CT angiography show absence of hepatic arterial flow; transfer to cath lab for drug-eluting stent placement.	Continuous DAPT (aspirin 100 mg + clopidogrel 75 mg QD) initiated; subcutaneous unfractionated heparin (5,000 IU q8h) started for DVT prophylaxis.	Catheter maintained; patient-controlled epidural analgesia (PCEA) pump initiated in the ICU.
Days 1-4	Stable clinical and hemodynamical course.	DAPT (aspirin + clopidogrel) and subcutaneous unfractionated heparin (5,000 IU q8h) maintained.	Continuous PCEA pump maintained.
Day 5	Uncomplicated clinical evolution.	Unfractionated heparin transitioned to low-molecular-weight heparin (enoxaparin 40 mg subcutaneously once daily). DAPT maintained.	Continuous PCEA pump maintained.
Day 7	Identification of localized inflammatory signs at the epidural insertion site.	DAPT and enoxaparin maintained.	PCEA pump discontinued; multidisciplinary consensus convened.
Day 8	Pre-removal laboratory workup performed.	Strict 24-hour withholding period of both DAPT and enoxaparin completed.	Extraction of the thoracic epidural catheter.
Day 8 (Post-removal)	Immediate initiation of rigorous neurological monitoring.	Aspirin and clopidogrel reintroduced at two hours post-removal; enoxaparin resumed at six hours post-removal.	Catheter successfully removed.
Days 9-10	Neurological monitoring maintained for 48 hours (q2h for the first 24h, then q4h); fully preserved neurological function; hospital discharge in good condition.	DAPT and enoxaparin maintained at therapeutic/prophylactic doses.	Discharged without complications.

The prolonged retention of the epidural catheter presented a clinical dilemma, as the concurrent anticoagulation and antiplatelet regimen precluded its removal within the safety intervals recommended by ASRA guidelines. Following the onset of inflammatory signs at the catheter insertion site on the seventh postoperative day, laboratory evaluation revealed a hemoglobin of 8.5 g/dL, hematocrit of 24.5%, activated partial thromboplastin time (aPTT) of 24.9 seconds (ratio 0.89), prothrombin time of 13.8 seconds (international normalized ratio (INR) 1.20, activity 76%), platelet count of 209,000/mm³, fibrinogen of 716 mg/dL, and ionized calcium of 4.8 mg/dL. However, viscoelastic testing, such as rotational thromboelastometry (ROTEM), and advanced qualitative platelet function assays were unavailable. Faced with this scenario, a multidisciplinary team - comprising anesthesiology, hematology, vascular surgery, and surgical oncology - convened to weigh the risks and benefits. A consensus was reached to remove the catheter following a strict 24-hour withholding period for both DAPT and LMWH.

Aspirin and clopidogrel were reintroduced two hours after catheter extraction, followed by the resumption of LMWH at six hours post-removal. Rigorous neurological monitoring was initiated immediately upon catheter removal and maintained for 48 hours to promptly identify any signs of a spinal hematoma. Assessments included examination of lower extremity motor strength, tactile and pain sensation, sphincter function, and investigation of axial or radicular pain. Initially, evaluations were performed every two hours for the first 24 hours, and every four hours thereafter. Throughout this monitoring period, the patient maintained muscle strength grade 5/5 in all lower extremity muscle groups, preserved sensation, absence of radicular pain, and normal sphincter function. No signs of neurological compromise were identified, and the patient was discharged from the hospital in good condition.

## Discussion

The incidence of epidural hematoma varies widely depending on the patient population and clinical context, with an estimated rate of 1 in 150,000 epidural anesthetics [4]. Although rare, this complication is potentially catastrophic. Hematomas may develop during both catheter insertion and removal, with current literature indicating a higher risk associated with the latter [5]. Complete neurological recovery is uncommon following the onset of a symptomatic hematoma; therefore, early recognition and prompt surgical intervention are paramount to optimize outcomes [1]. To mitigate the risk of neuraxial bleeding, the latest guidelines from the ASRA establish strict safety intervals for epidural catheter manipulation in patients receiving antithrombotic and antiplatelet therapy (Table 2).

**Table 2:** Summary of ASRA recommendations for epidural catheter placement and removal in patients receiving antithrombotic and antiplatelet therapy ASRA: American Society of Regional Anesthesia and Pain Medicine; ASA: acetylsalicylic acid; UFH: unfractionated heparin; aPTT: activated partial thromboplastin time; anti-Xa: anti-factor Xa Adapted from reference [3] under the terms of the Creative Commons Attribution (CC BY) license.

Drug/dose	Interval between the last dose and neuraxial block placement	Interval between the last dose and catheter removal	Timing of next dose after catheter removal
Aspirin (ASA)	No restriction as monotherapy. Caution is advised with concurrent medications affecting coagulation.	No restriction as monotherapy. Caution is advised with concurrent medications affecting coagulation.	No restriction
Clopidogrel	Discontinue five to seven days prior	Discontinue five to seven days prior (catheter should not be maintained in patients on active therapy)	Immediate (without loading dose). Wait > 6 hours (with loading dose)
Prophylactic Enoxaparin (e.g., 40 mg/day)	Wait ≥ 12 hours	Wait ≥ 12 hours	Wait ≥ 4 hours
Therapeutic Enoxaparin (e.g., 1 mg/kg every 12h)	Wait ≥ 24 hours	Wait ≥ 24 hours	Wait ≥ 4 hours
Intravenous UFH	Discontinue for four to six hours (assess aPTT/Anti-Xa)	Discontinue for four to six hours (assess aPTT/anti-Xa)	Wait ≥ 1 hour
Low-dose subcutaneous UFH (e.g., 5,000 IU every 8h)	Wait ≥ 4-6 hours	Wait ≥ 4-6 hours	Immediately (anticoagulant effect only after 40-60 minutes)

In the present case, strictly adhering to these evidence-based guidelines proved clinically unfeasible. While ASRA guidelines confirm that maintaining aspirin monotherapy is safe during any epidural manipulation, this safety profile does not extend to DAPT scenarios. Under standard recommendations, clopidogrel requires a five- to seven-day discontinuation period prior to catheter removal, alongside a mandatory 12-hour waiting period following the last dose of prophylactic enoxaparin [3]. A prolonged interruption of DAPT in a patient with a recently deployed drug-eluting stent in the hepatic artery was precluded, as it would likely precipitate acute stent thrombosis, ischemia, and failure of the hepatic remnant.

Concurrently, retaining the epidural catheter in the presence of active inflammatory signs posed an imminent risk of a central nervous system infection, such as an epidural abscess or meningitis. Consequently, navigating this conflict between established neuraxial guidelines and the patient's acute ischemic and infectious risks required a departure from standardized protocols, relying instead on multidisciplinary expert consensus.

An unorthodox 24-hour simultaneous withholding window for both antiplatelet therapy and enoxaparin was established prior to catheter removal. A review of the scarce literature reveals that successful epidural catheter removals in patients on DAPT have utilized similarly abbreviated clopidogrel cessation windows (ranging from 24 to 72 hours); however, these instances relied heavily on advanced point-of-care viscoelastic testing (e.g., ROTEM, thromboelastography (TEG)) or platelet function assays to objectively confirm adequate hemostasis prior to manipulation [4,6,7]. Conversely, catheter removal under active antithrombotic therapy without adequate cessation or compensatory strategies has been associated with catastrophic spinal epidural hematoma and permanent paraplegia [8].

In the present case, the unavailability of advanced viscoelastic testing precluded the objective assessment of residual clopidogrel inhibition. To navigate this technical limitation, the standard 12-hour withholding period for prophylactic enoxaparin was deliberately extended to 24 hours. This provided a vital clinical compensatory safety margin, aiming to ensure complete clearance of anti-factor Xa (anti-Xa) activity to counteract the bleeding risk imposed by the anticipated residual platelet dysfunction. Finally, to mitigate the risk of acute stent thrombosis following removal, drug reintroduction was performed in an early and staggered manner: aspirin and clopidogrel were restarted two hours after catheter removal, followed by enoxaparin at six hours.

Serial neurological monitoring over the subsequent 48 hours confirmed the success of this strategy, with no evidence of neuraxial hematoma. However, it is imperative to acknowledge the limitations of this report. This successful outcome represents a highly individualized, salvage decision-making process tailored to a unique patient with competing life-threatening priorities. It must not be interpreted as a safe alternative to, or a justification for deviating from, current ASRA recommendations. In the absence of formal guidelines for such exceptional scenarios, interdisciplinary communication, individualized risk stratification, and rigorous postoperative neurological monitoring remain paramount.

## Conclusions

Epidural catheter removal in the setting of concurrent DAPT and prophylactic anticoagulation contravenes current ASRA safety guidelines due to the elevated risk of neuraxial hematoma. However, this report illustrates that exceptional clinical scenarios - such as the overlapping risks of critical arterial stent thrombosis and central nervous system infection - require highly individualized, multidisciplinary decision-making. While the implementation of an unorthodox 24-hour compensatory withholding window proved successful for this specific patient, this salvage strategy must not be interpreted as an alternative to, or justification for deviating from, established ASRA recommendations. Given the inherent limitations of a single case report, this approach cannot be generalized. Further robust evidence is urgently required to safely guide the management of neuraxial catheters in patients receiving complex antithrombotic regimens. In the absence of such data, rigorous risk-benefit assessments and vigilant postoperative neurological monitoring remain paramount.
